# Pattern of anticoagulation prescription for elderly atrial fibrillation patients with or without severe dementia: A retrospective analysis of patient data

**DOI:** 10.1097/MD.0000000000042343

**Published:** 2025-05-09

**Authors:** Abdulrahman M. AlTuraiki, Haya M. AlMalag, Shahad M. AlShehri, Jumanah M. AlKendi, Alanoud M. AlAnazi, Dalal A. AlAbdulkarim, Shiekha S. AlAujan

**Affiliations:** a Department of Pharmaceutical Care, Ministry of the National Guard – Health Affairs, King Abdullah International Medical Research Center, King Saud bin Abdulaziz University for Health Sciences, Riyadh, Saudi Arabia; b Department of Clinical Pharmacy, College of Pharmacy, King Saud University, Riyadh, Saudi Arabia.

**Keywords:** anticoagulant, atrial fibrillation, dementia, elderly, geriatric, Saudi Arabia

## Abstract

Atrial fibrillation (AF) is a very common type of cardiac arrhythmia. Use of an anticoagulant is highly recommended. We aimed to identify the pattern of prescribing of an oral anticoagulant (OA) in patients with AF and severe dementia or patients with AF aged > 80 years. A retrospective review of medical charts was conducted in 2 tertiary care centers in Riyadh, Saudi Arabia: King Saud University Medical City and King Abdulaziz Medical City. Data for people with AF retrieved between January 2016 and December 2020 from hospital information systems. Collected data included demographics, medical history, medication history (including use of an OA or antiplatelet agent), stroke and major bleeding history. Adjusted binary logistic regression was used to predict the odds ratio (OR) of the primary outcome and secondary outcomes. The data of 620 patients were assessed. Most (60%) were women. The average age of study cohort was 79 ± 6.1 years. Most patients (88.2%) were prescribed an OA. The most commonly prescribed OA was a direct inhibitor of factor Xa (DIFXa; 48%), followed by a coumarin derivative (36%), and direct inhibitor of thrombin (16%). Patients using a coumarin derivative carried higher OR of developing severe dementia (adjusted OR = 2.687, 95%CI = 1.795–4.021, *P*-value < .001). Most patients suffering from AF were prescribed an OA. A DIFXa inhibitor was the most prescribed OA. Use of a coumarin derivative carried a high prevalence of dementia among our study cohort.

## 1. Introduction

Atrial fibrillation (AF) is the most common type of cardiac arrhythmia.^[1]^ About 33.5 million people worldwide had AF in 2010.^[2]^ AF prevalence is increasing with age, and will affect 6 to 12 million people in the United States of America by 2050 and 17.9 million in Europe by 2060.^[3–5]^

AF is associated with 5-fold increased risk of ischemic stroke.^[6]^ Patients with dementia carry a high risk of suffering a stroke.^[7]^ Two studies in the central and western regions of Saudi Arabia (SA) showed diabetes mellitus, hypertension, and ischemic heart disease to be the most common risk factors for AF in the central region of SA. However, increased age in the western region of SA was the main cause of AF.^[8,9]^ Another study In SA suggested rheumatic valvular disease to be the most common cause of AF, and arterial thromboembolism to be the most serious complication of AF.^[10]^

By using adjusted-dose warfarin or antiplatelet agents, the risk of stroke can be reduced by approximately 60% and 20%, respectively, in patients with AF.^[10]^ Oral anticoagulants (OA) is a standard of care in patients with high CHA_2_DS_2_-VASc score ≥ 2.^[11]^ Warfarin is the most commonly prescribed anticoagulant.^[12]^ Several direct OAs (DOACs), including apixaban, dabigatran, rivaroxaban, and edoxaban, have been approved by the US Food and Drug Administration for the prevention of stroke and systemic emboli for patients with nonvalvular AF.^[12]^ DOACs are as efficacious as warfarin in preventing ischemic stroke in patients with AF, and are also associated with lower risk of hemorrhage.^[13,14]^

Around 10% of “older” (>65 years) people will be affected by some degree of dementia and around 30% of “geriatric” (>85 years) people may have the signs or symptoms of dementia.^[15,16]^ Old age and dementia are associated with hemorrhagic complications for several reasons (e.g., risk of falling, and polypharmacy). Such complications can lead to different interactions with, and may change the metabolism of, warfarin. Hence, older patients with dementia are less likely to receive warfarin.^[17]^

Because of these limitations, controversy rages about the use of anticoagulation in patients suffering from dementia. Guidelines from the American Academy of Neurology for stroke prevention in AF state that data are insufficient to determine whether anticoagulants are safe or effective in elderly patients who have frequent falls or advanced dementia.^[18]^ Guidelines set by the European Stroke Organization recommend using anticoagulants in high-risk older patients with cognitive deficits, but the level of evidence for such a recommendation is weak.^[19]^ Dementia is not a contraindication for anticoagulation unless compliance cannot be guaranteed according to guidelines set by the European Society of Cardiology.^[20]^

Studies in SA on the patterns of anticoagulant prescribing in AF patients aged > 65 years with severe dementia, or in patients aged > 80 years, are lacking. Therefore, we aimed to identify the pattern of prescribing of OAs in these groups.

## 2. Methods

### 2.1. Ethical approval of the study protocol

The study protocol was approved by the Ethics Review Board of King Saud University Medical City (KSUMC; E-19-4110) and King Abdulaziz Medical City (RC19/270/R) in Riyadh, SA.

### 2.2. Study design

This was a retrospective cohort study of AF participants aged > 80 years, or aged > 65 years with severe dementia. It was conducted in KSUMC and King Abdulaziz Medical City. Information on all patients diagnosed with AF and aged > 65 years was retrieved from the information systems of these 2 hospitals. This information was screened against the inclusion criteria. The study was conducted between January 2016 until December 2020. The study was guided by the Strengthening the Reporting of Observational Studies in Epidemiology (STROBE) checklist for a cohort study design.^[21]^ Informed consents were not needed as the study was retrospective design with no patients’ identifiers extracted.

### 2.3. Participants

All patients diagnosed with AF were included in the study if they were aged > 80 years or if they were aged > 65 years and had AF and with a diagnosis of severe dementia according to The Global Deterioration Scale for Assessment of Primary Degenerative Dementia.^[22]^ No specific exclusion criteria were considered.

### 2.4. Variables

We collected information on: age, sex, and body mass index at baseline; estimated glomerular filtration rate (eGFR); medical history; use of OAs (direct inhibitor of factor Xa (DIFXa; rivaroxaban and apixaban); direct inhibitor of thrombin (DIT; dabigatran); coumarin derivative (warfarin)), antiplatelet agents, or other medications; history of major bleeding, blood transfusion, or ischemic stroke.

### 2.5. Endpoints

The study primary endpoint was the prevalence of prescribed OAs to AF patients. The secondary endpoints were: the difference in demographics of patients receiving an OA vs those who did not receive an OA; identification of the most commonly prescribed OA; prevalence of bleeding and stroke complications among patients who received different OAs.

### 2.6. Study size

Peduzzi et al recommended the following formula for calculating the minimum number of samples for estimation of logistic regression^[23]^:

Minimum number of samples = (N = 10 k/p)

where p is the smallest number of people in the population with dementia (30%) and k is the number of independent variables, which was 10 variables in the current study. Hence, the calculation would be N = (10 × 10)/(0.3). The minimum number of cases required was 333.

### 2.7. Statistical analyses

Variables were coded and entered into SPSS 27.0 (Chicago).^[24]^ Categorical variables are displayed as numbers and percentages. Continuous variables are displayed as the mean ± SD, or median and interquartile range (IQR). Comparative tests included the independent-sample *t*-test, nonparametric Mann–Whitney *U*-test, and chi-squared test based on the type of data. Binary logistic regression was used to predict unadjusted odds ratios (ORs) and adjusted ORs (AORs) of primary and secondary variables across groups taking into account confounders. *P*-value < .05 was considered significant. Complete case analysis is used for missing data.

## 3. Results

A total of 620 files of eligible patients suffering from AF with or without an OA were included in our analyses. The entire study cohort had a mean age of 79 ± 6.1 years. Most of the study cohort was women (59.6%) and had a mean body mass index of 29.9 ± 7.3 kg/m^2^. An antiplatelet was used in 45.3% of the study cohort. Small percent of the patients (1.5%) were using either strong CYP3A4 inducer or inhibitor. Severe dementia was present in 36% of participants. Comorbidities other than dementia were hypertension (87.6%), diabetes mellitus (60.6%), heart failure (42.9%), and ischemic heart disease (31.3%). With regard to secondary-outcome variables, 18% had a history of bleeding and 22.3% had a history of ischemic stroke. Of the 620 participants, the majority of the study sample were on OA (n = 547, 88.2%). When data stratified according to OA use, nonsignificant difference were found in patients characteristics between groups, except for hypertension (*P*-value < .001), and heart failure comorbidities (*P*-value = .036) were higher in OA users group compared to OA non-users group (Table 1). Also, the use of antiplatelet agent (*P*-value = .006) was lower (34.3%) in OA users group compared to OA non-users group (Table 1).

**Table 1:** Participants characteristics, stratified by the use of OAs.

		Total (N = 620)	Non-users of OAs (N = 73 [11.8%])	Users of OAs (N = 547 [88.2%])	*P*-value
Age, yr, mean (SD)		79.0 (6.1)	79.1 (6.9)	79.0 (6.0)	.882
Sex, N (%)	Male	248 (40.4)	24 (34.3)	224 (41.2)	.302
	Female	366 (59.6)	46 (65.7)	320 (58.8)	
Body mass index, kg/m^2^, mean (SD)		29.9 (7.3)	28.9 (9.1)	30.1 (7.0)	.207
Use of antiplatelet, N (%)		281 (45.3)	44 (60.3)	**237 (43.3**)	**.006** *
Use of strong CYP3A4 inhibitor, N (%)		9 (1.5)	1 (1.4)	8 (1.5)	.712
Use of strong CYP3A4 inducer, N (%)		9 (1.5)	0 (0.0)	9 (1.6)	.321
Severe dementia, N (%)		223 (36.0)	19 (26.0)	204 (37.4)	.058
Hypertension, N (%)		543 (87.6)	54 (74.0)	**489 (89.4**)	**<.001** *
Diabetes mellitus, N (%)		375 (60.6)	37 (50.7)	338 (61.9)	.065
Ischemic heart disease, N (%)		194 (31.3)	16 (21.9)	178 (32.5)	.066
Peripheral vascular disease, N (%)		34 (5.5)	5 (6.8)	29 (5.3)	.372
Heart failure, N (%)		266 (42.9)	23 (31.5)	**243 (44.4**)	**.036** *
Thyroid disorder, N (%)		107 (17.5)	12 (11.2)	95 (88.8)	.838
Chronic viral hepatitis, N (%)		26 (4.2)	4 (5.5)	22 (4.0)	.368
Malignancy, N (%)		30 (4.8)	7 (9.6)	23 (4.2)	.052
Liver cirrhosis, N (%)		25 (4.0)	3 (4.1)	22 (4.0)	.583
History of bleeding, N (%)		111 (18.0)	14 (19.2)	97 (17.8)	.773
History of stroke, N (%)		138 (22.3)	16 (21.9)	122 (22.3)	.934

Bold indicates significantly higher percentage.

OA = oral anticoagulant.

* *P*-value < .05.

**Figure 1.**
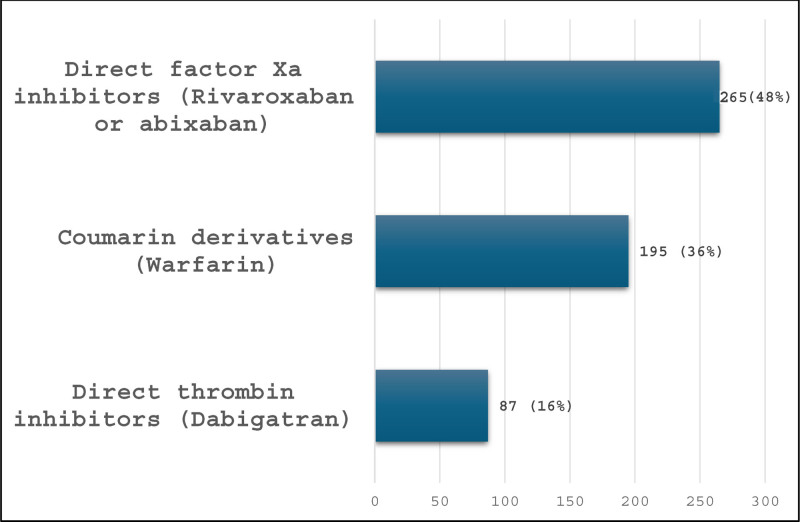
Most commonly used OA (n = 547). OA = oral anticoagulant.

In regard to duration of OAs use, 88% (n = 547) of patients received an OA for a median (IQR) duration of 1054 (956–1120) days. The most commonly used OAs were a DIFXa (48%), followed by a coumarin derivative (36%), and dabigatran (16%) (Fig. 1). Table 2 shows stratified data based on the type of OA used. Patients who used a coumarin derivative had a significantly higher prevalence of dementia (50.3%, *P*-value < .001) and a significantly longer duration of use of an OA (median (IQR) of 1191 (1156–1246) days, *P*-value < .001). The eGFR at baseline was significantly higher (>90 mL/min/1.73 m^2^) in people taking a DIFXa (*P*-value < .001).

**Table 2:** Variables of primary and secondary outcomes with contributing factors stratified by type of OA.

		Total (N = 620)	Non-users of OAs (N = 73 [11.8%])	Direct inhibitor of thrombin (dabigatran) (N = 87 [14%])	Direct inhibitor of factor Xa (rivaroxaban or apixaban) (N = 265 [42.7%])	Coumarin derivative (warfarin) (N = 195 [31.5%])	*P*-value
Severe dementia, N (%)		223 (36.0)	19 (26.0)	2 (2.3)	104 (39.2)	**98 (50.3**)	<.001*
History of bleeding, N (%)		111 (18.0)	14 (19.2)	19 (21.8)	44 (16.6)	34 (17.4)	.716
History of ischemic stroke, N (%)		138 (22.3)	16 (21.9)	16 (18.4)	58 (22.0)	48 (24.6)	.709
Duration of anticoagulant use, days, median (IQR)		1054 (956 – 1120)	-	895 (548 – 1115)	1048 (919 – 1135)	**1191 (1156 – 1246**)	<.001*
Baseline ALT upon starting oral anticoagulant	Low	66 (10.7)	9 (12.7)	7 (8.0)	33 (12.5)	17 (8.7)	.473
	Within normal limit	546 (88.1)	60 (82.2)	80 (92.0)	228 (86.0)	176 (90.3)	
	High	7 (1.1)	2 (2.7)	0 (0.0)	3 (1.1)	2 (1.0)	
Baseline eGFR in ml/min/1.73 m^2^ before starting anticoagulant	<15 or on hemodialysis	21 (3.4)	3 (4.1)	1 (1.1)	4 (1.5)	13 (6.7)	<.001*
	15 to 29	27 (4.4)	5 (6.8)	0 (0.0)	5 (1.9)	17 (8.7)	
	30 to 59	193 (31.1)	22 (30.1)	28 (32.2)	77 (29.1)	66 (33.8)	
	60 to 89	255 (41.1)	26 (35.6)	43 (49.4)	112 (42.3)	71 (36.4)	
	>90	123 (19.8)	15 (20.5)	15 (17.2)	**66 (24.9**)	27 (13.8)	
History of blood transfusion, N (%)		28 (4.5)	6 (8.2)	6 (6.9)	11 (4.2)	5 (2.6)	.140
History of 2-g drop in hemoglobin, N (%)		129 (20.8)	17 (23.3)	21 (24.1)	56 (21.1)	35 (17.9)	.551

Bold indicates significantly higher percentage or value.

ALT = alanine aminotransferase, eGFR = estimated glomerular filtration rate, IQR = interquartile range, OA = oral anticoagulant.

* Statistically significant (*P*-value < .05).

**Figure 2.**
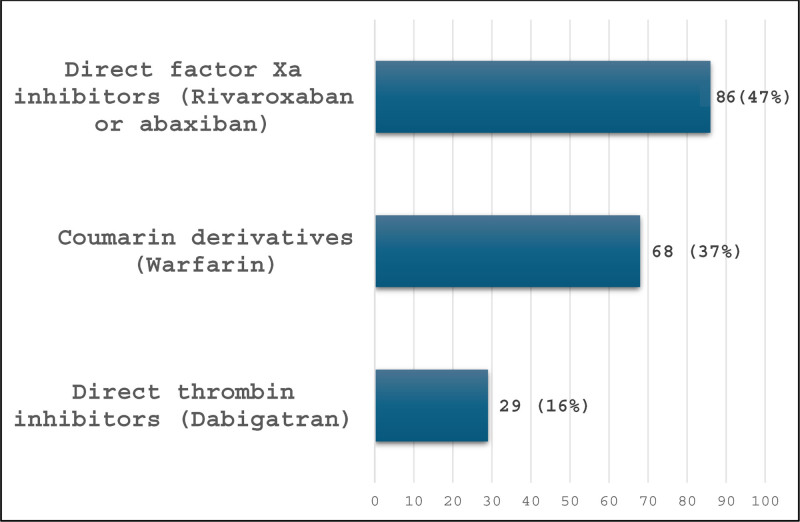
Number of bleeding or stroke events according to class of OA used (n = 183). OA = oral anticoagulant.

According to binary logistic regression (Table 3), nonsignificant unadjusted and adjusted ORs were found when using OAs and the presence of severe dementia, history of bleeding, or ischemic stroke. Participants using a DIT had a lower chance of suffering severe dementia (OR = 0.033, 95%CI = 0.008–0.135, *P*-value < .001; AOR = 0.035, 95%CI = 0.008–0.147, *P*-value < .001). Participants using a coumarin derivative had a higher odds of having severe dementia even after adjustments of confounders (OR = 2.434, 95% CI = 1.714–3.455, *P*-value < .001; AOR = 2.687, 95%CI = 1.795–4.021, *P*-value < .001). The other variables we tested were not significantly different between groups. Although not significant, most events of bleeding or stroke were with use of a DIFXa (47%), followed by a coumarin derivative (37%), and a DIT (14%) (Fig. 2).

**Table 3:** Use of an OA and risk of dementia, bleeding, and stroke using adjusted and unadjusted binary logistic regression.

	OR	95% CI	*P*-value	AOR†	95% CI	*P*-value
Use of oral anticoagulant						
Severe dementia	1.695	0.977 – 2.941	0.060	1.730	0.968 – 3.091	.064
History of bleeding	0.912	0.490 – 1.701	0.773	0.877	0.469 – 1.642	.683
History of ischemic stroke	1.025	0.568 – 1.849	0.934	0.986	0.544 – 1.789	.964
Subgroup analysis of variables of primary and secondary outcomes according to the class of oral anticoagulant						

	OR	95% CI	*P*-value	AOR‡	95% CI	*P*-value
Direct inhibitor of thrombin (dabigatran)						
Severe dementia	0.033	0.008 – 0.135	<0.001*	0.035	0.008 – 0.147	<.001*
History of bleeding	1.327	0.761 – 2.314	0.318	1.231	0.690 – 2.196	.482
History of ischemic stroke	0.754	0.422 – 1.345	0.338	0.788	0.434 – 1.433	.435
Direct inhibitor of factor Xa (rivaroxaban or apixaban)						
Severe dementia	1.265	0.908 – 1.771	0.164	1.200	0.826 – 1.742	.339
History of bleeding	0.851	0.560 – 1.294	0.450	0.820	0.530 – 1.267	.371
History of ischemic stroke	0.961	0.655 – 1.410	0.838	0.971	0.649 – 1.453	.887
Coumarin derivative (warfarin)						
Severe dementia	2.434	1.714 – 3.455	<0.001*	2.687	1.795 – 4.021	<.001*
History of bleeding	0.952	0.610 – 1.486	0.829	0.883	0.551 – 1.416	.605
History of ischemic stroke	1.205	0.807 – 1.798	0.362	1.208	0.783 – 1.865	.393

AOR = adjusted odds ratio, CI = confidence interval, OA = oral anticoagulant, OR = odds ratio.

† Adjusted to age and sex.

‡ Adjusted to age, sex, duration of anticoagulant use and estimated glomerular filtration rate at baseline.

* Statistically significant (*P*-value < .05).

## 4. Discussion

A high pattern of prescribing OAs in older patients with AF was observed in the study. Approximately one-third of our study cohort was diagnosed with severe dementia.

Hypertension is a very common comorbidity. The prevalence of hypertension in our cohort was slightly higher (87%) than that documented in another study, which have reported 60% to 80% of patients with AF suffering hypertension.^[25]^

The most commonly prescribed OA group was a DIFXa (apixaban or rivaroxaban), followed by a coumarin derivative, and dabigatran. The low use of dabigatran in the present study could have been due to the increased prevalence of bleeding in older patients reported in the literature, especially at a high dose (150 mg, twice daily).^[26]^ The use of an antiplatelet agent was very common in our study population, and was higher in the group of patients not taking an OA.

Our study showed that clinicians were more adherent to guidelines for prescribing OAs than those in study conducted in Thailand.^[17]^ In the current study, 88% of the study cohort were prescribed an OA. This figure is higher than Krittayaphong and colleagues (81%) study.^[27]^ Our study cohort was older and carried a higher risk of bleeding. Hence, clinicians in SA had a high level of adherence for prescribing an OA in a higher-risk group.

Several studies have indicated that use of an antiplatelet agent is associated with a lower OR of prescribing an OA.^[27,28]^ This phenomenon was also observed in our study.

A higher prevalence of dementia among patients receiving a coumarin derivative was found compared with that in the other groups. Due to our study design, this finding may not establish causation even after adjustment of confounders. Nevertheless, taking a coumarin derivative was associated with higher OR of suffering severe dementia. In a large study based on the matching of adjusted propensity scores,^[29]^ using an OA was associated with a lower risk of developing dementia compared with not using an OA. Also, users of a DOAC have been shown to carry a lower risk of developing dementia compared with patients using warfarin.^[29]^ This finding was in line with a meta-analysis of 9 studies, DIFXas (rivaroxaban and apixaban) were found to be associated with a lower risk of developing dementia compared with warfarin.^[30]^ Other study have shown the risk of dementia with warfarin among patients with an uncontrolled International Normalized Ratio.^[31]^ In 2 randomized clinical trials, a lower prevalence of intracranial bleeding were found in users of a DIFXa compared with those using warfarin, and this may explain the lower prevalence of dementia in groups using a DIFXa.^[13,14]^ This finding requires further assessment in a larger trial with a more robust design to establish the risk of warfarin use leading to dementia compared with that of other medications.

The main limitation in our study was that we did not assess the quality of anticoagulation among patients receiving warfarin or the appropriateness of the doses for patients receiving other OAs. A causal relationship between a certain OA and risk of developing bleeding or ischemic stroke were not assessed because the data were obtained from hospital information systems, which may have missed instances of bleeding or stroke. Finally, the generalisability of the study results were limited to the study setting.

## 5. Conclusions

A high prevalence of OA prescription in older patients with AF in SA was observed. DIFXas were the most prescribed OA. Warfarin use was associated with a high prevalence of severe dementia. Dementia showed a high prevalence in our study cohort.

## Acknowledgments

The authors thanks the Researchers Supporting Project at King Saud University for the financial support.

## Author contributions

**Conceptualization:** Abdulrahman M. AlTuraiki.

**Data curation:** Abdulrahman M. AlTuraiki, Haya M. AlMalag, Shahad M. AlShehri, Jumanah M. AlKendi, Alanoud M. AlAnazi, Dalal A. AlAbdulkarim, Shiekha S. AlAujan.

**Funding acquisition:** Shiekha S. AlAujan.

**Methodology:** Abdulrahman M. AlTuraiki.

**IRB Approval:** Abdulrahman M. AlTuraiki, Shiekha S. AlAujan.

**Project administration:** Abdulrahman M. AlTuraiki, Shiekha S. AlAujan.

**Software:** Haya M. AlMalag.

**Formal analysis:** Haya M. AlMalag.

**Supervision:** Abdulrahman M. AlTuraiki, Shiekha S. AlAujan.

**Visualization:** Shiekha S. AlAujan.

**Writing – original draft:** Abdulrahman M. AlTuraiki, Haya M. AlMalag, Shiekha S. AlAujan.

**Writing – review & editing:** Abdulrahman M. AlTuraiki, Haya M. AlMalag, Shahad M. AlShehri, Jumanah M. AlKendi, Alanoud M. AlAnazi, Dalal A. AlAbdulkarim, Shiekha S. AlAujan.
